# Properties of Dried Apricots Pretreated by Ultrasound-Assisted Osmotic Dehydration and Application of Active Coatings

**DOI:** 10.17113/ftb.58.03.20.6471

**Published:** 2020-09

**Authors:** Roghieh Sakooei-Vayghan, Seyed Hadi Peighambardoust, Javad Hesari, Maral Soltanzadeh, Donatella Peressini

**Affiliations:** 1 Department of Food Science, College of Agriculture, University of Tabriz, 29^th^ Bahman Blvd., 5166616471 Tabriz, Iran; 2 Department of Agricultural, Food, Environmental and Animal Sciences, University of Udine, via Sondrio 2/A, 33100 Udine, Italy

**Keywords:** apricot, hot air drying, osmotic dehydration, ultrasound-assisted osmotic dehydration, active coating, physical properties

## Abstract

**Research background:**

The worldwide demand for healthy and sulphur-free dried vegetables and fruits has grown. Combined ultrasound-assisted osmotic dehydration (UOD) and application of active coatings incorporating natural preservatives represents an attractive alternative to sulphuring to preserve the sensorial and nutritional quality of dried fruits. The aim of this study is to investigate the effect of osmotic dehydration (OD) and UOD, and the use of pectin coatings (alone or with citric acid or ascorbic acid) on physical, textural and microstructural properties of hot air-dried apricots.

**Experimental approach:**

Fresh apricot cubes (1 cm^3^) were pretreated with either OD at 55 °C for 30 and 45 min or UOD at two ultrasonic frequencies of 25 and 35 kHz for 30 and 45 min followed by application of active coatings with pectin alone, pectin with citric acid or pectin with ascorbic acid for 10 min. All pretreated coated samples were then hot air-dried at 60 °C until a final moisture content of 20% (wet basis) was reached. Physical (shrinkage, apparent and bulk densities), chemical (browning value and water activity) and textural properties (firmness and shrinkage), microstructure and microbial load of dried apricots were studied.

**Results and conclusions:**

Application of OD and UOD improved physical and textural properties of the dried apricots. Moreover, apparent and bulk densities, rehydration capacity of OD and UOD pre-treated samples increased, while shrinkage, water activity and microbial load decreased. Firmness of UOD pretreated samples was significantly (p<0.05) lower than that of OD ones. Likewise, increasing ultrasound frequency from 25 to 35 kHz led to a significant decrease in *F*_max_ values of dried apricots. Furthermore, coating of the processed samples with pectin and citric acid increased *F*_max_ value and decreased rehydration capacity of dried apricots. Scanning electron microscopy of both OD and UOD samples illustrated improvement of textural properties. The utilization of both OD pretreatment and edible pectin coatings resulted in a decrease in browning values. However, UOD increased browning values of the dried apricots. Coating of UOD samples with pectin and ascorbic acid resulted in substantial discolouration in hot air-dried apricots.

**Novelty and scientific contribution:**

This study advances the knowledge in the field of fruit drying by combined application of OD or UOD pretreatments with active edible coatings on different properties of hot air-dried apricots.

## INTRODUCTION

Nowadays, there is a growing demand for healthy and nutritive foods. Apricot contains a high content of polyphenolic compounds, carotenoids, minerals and vitamins, which are nutritionally valuable compounds (*1*). Nutritional content in fruits and vegetables such as apricot not only depends on size, variety and ripeness (*2*), but also on processing conditions (*3*). The small amounts of apricot are consumed fresh, and its processing is necessary to extend its shelf life. Hot air drying is most common process to increase the shelf life of fruits, but it causes irreversible changes in nutritional and physical properties of apricots such as colour and textural variations and decreases nutritional value (*3*). Textural damages created in air-dried fruits and vegetables include extreme shrinkage, low rehydration capacity, and texture firmness. Colour changes in hot air-dried apricot drives from ascorbic acid oxidation, enzymatic and non-enzymatic browning reactions. Sulphur dioxide is normally used as a synthetic antioxidant before drying to preserve the colour and to protect carotenoids, polyphenolic compounds and vitamin C (*4*, *5*). However, its use in fresh fruits and vegetables is restricted by Food and Drug Administration (FDA) regulations because of its role in the initiation of asthmatic reactions in sensitive people.

Osmotic dehydration (OD) pretreatment can substitute sulphite application before drying. This process improves nutritional value, texture properties, reduces shrinkage, and prevents colour deterioration during the drying of fruits and vegetables (*6*). Concerning low mass transfer rate in the OD, the use of high-power ultrasound can enhance mass transfer rate of the process (*7*). Combining power ultrasound in OD processing creates cavities in the liquid phase and enhances the rate of mass transfer by forming micro agitation and reducing the thickness of the solid diffusion boundary layer. In the solid phase, alternating compressions and expansions result in a sponge-like effect and create microchannels that facilitate the flow of water out of the solid medium (*8*).

Coating of fruits and vegetables with edible carbohydrate-based coatings before drying is another pretreatment that can decrease oxidation and nutritional compound loss during hot air drying. Furthermore, coatings can minimize colour changes in the dried materials due to gas barrier properties (*9*). Garcia *et al*. (*10*) reported that the application of edible coatings on papaya before drying increased the retention of vitamin C content compared with non-coated dried papaya. Moreover, Silva *et al*. (*9*) found that coating of pineapple samples with pectin, and whey protein isolate and locust bean gum coatings decrease the loss of vitamin C content in coated samples during drying. They showed that the lowest colour change among samples happened in the pectin-coated ones. Ghasemzadeh *et al*. (*11*) found that the use of pectin coating on raisin before drying resulted in better colour, flavour and texture.

There are many published papers about the use of ultrasound-assisted osmotic dehydration (UOD) and its effect on quality parameters of fruits and vegetables. To the best of our knowledge, no study has been done combining UOD and the application of active edible coatings. Thus, the novelty of the paper is the combined application of UOD and active edible coatings with different antioxidant agents before hot air drying. We investigated the effect of such processes on physical (shrinkage, apparent and bulk densities), chemical (browning value and water activity) and textural properties (maximum force and shrinkage), microstructure and microbial properties of dried apricots.

## MATERIALS AND METHODS

### Chemicals

Liquid sorbitol (70 °Brix) (Foodchem, Shanghai, PR China) was used as osmotic solution. Low methylated amidated pectin (GRINDSTED® LA210, the degree of methoxylation=0.44, degree of amidation=0.18; DANISCO, Copenhagen, Denmark), ascorbic acid (Northeast Pharmaceutical, Shenyang, PR China) and citric acid (Union Biochemical Co., Yixing, PR China) were used for the preparation of polysaccharide-based active edible coatings. Glycerol (Sigma-Aldrich, Merck, Munich, Germany) served as plasticizer agent.

### Fruit sample preparation

Fresh apricots (*Prunus armeniaca*) cultivar Harostar (formerly HW 436) from Asgarabad, Iran, were directly taken from the Urmia (Iran) agricultural region and transported to the laboratory in wooden boxes. Mature fruits with average mass of 25 g and average diameter of 3 cm were selected. The apricots were refrigerated at 4 °C and 80-90% relative humidity for maximum seven days until they were used. The initial moisture content of the fruits was 80% (wet basis). Before each experiment, apricots were removed from the refrigerator and left to equilibrate to room temperature. They were then washed, halved, stoned and cut to 1 cm^3^ cubes with a household tool.

### Osmotic dehydration

The sliced cubes (1 cm^3^) of fresh apricots (100 g) were immersed in sorbitol solution (35 °Brix, 400 g) giving a mass ratio of fruit to osmotic solution 1:4. Osmotic dehydration (OD) was carried out at 55 °C for 30 and 45 min. To maintain this temperature constant, the glass beaker containing apricot cubes immersed in the osmotic solution was placed in a water bath kept at 55 °C. The process temperature was controlled by a thermometer during osmosis. Using high OD temperature ensured the inactivation of polyphenol oxidase enzyme, as confirmed by Cheng *et al.* (*12*). Short treatment times below 45 min also limit the solute uptake by fruit samples due to the fact that water loss to solid gain ratio stays high at the early stages of the process (*13*, *14*). OD-treated apricot cubes were removed from the sorbitol solution and their excess liquid was taken using an absorbent paper.

### Ultrasound-assisted osmotic dehydration

Fresh apricot cubes prepared under the conditions mentioned above for OD treatment were subjected to power ultrasound (HD 2070.2; Sonopuls Berlin, Germany) with an ultrasound intensity of 4.3 W/g. The probe was put in the centre of a glass beaker containing sorbitol solution and apricot cubes at the height of 25 mm from the base of the container. Two levels of 25 and 35 kHz ultrasonic frequencies were applied for 30 and 45 min. To avoid temperature fluctuation during ultrasound processing, ice packs were placed around the glass beaker containing samples and the process temperature was controlled by a thermometer. Osmotic solution was stirred every 2 min by a glass agitator to ensure a homogenous osmotic treatment. The fruits were removed from the sorbitol solution, and the excess osmotic solution was removed through an absorbent paper.

### Active coating application

Following OD or UOD treatments, apricot samples were coated using a solution of low methylated amidated pectin (2%) prepared according to the method explained by Garcia *et al.* (*10*). For this purpose, 0.2 g glycerol (as plasticizer) and 2 g citric or ascorbic acid (as antioxidants) were added to every 100 mL of pectin solution. Apricot cubes were dipped in pectin, pectin with citric acid or pectin with ascorbic acid solutions for 10 min, followed by rinsing and removing their excess coating liquid, and hot-air drying.

### Convective hot air drying

Apricot cubes were dried using a laboratory convective tray dryer (Armfield Ltd., Ringwood, Hampshire, UK) with a total capacity of approx. 3 kg using four sample trays, which were suspended from a digital balance mounted on the dryer top. The dryer was previously heated to the set-point temperature for about 30 min and then loaded with 0.4 kg (2 kg/m^2^) pretreated and coated apricot cubes. Samples were dried at 60 °C with an air velocity of 1.5 m/s. Changes of the sample mass during drying were continuously recorded until a final moisture content of 19-20% (wet basis) was reached (*15*).

### Moisture content measurement

The moisture content was estimated by vacuum drying (vacuum oven Vacutherm VT6025; Thermo Fisher Scientific Inc., Branchburg, NJ, USA) at 60 °C until a constant mass was reached (*16*).

### Soluble solids measurement

The total soluble solids of the liquid phase of the fruit (*X_s_*, in g soluble solids per g fruit liquid phase) were estimated by a refractometer (ATAGO, Pal ALFA, Tokyo, Japan). For fresh apricot, the liquid phase was obtained directly by pressing the fruit. In the case of the dried fruit, a controlled mass of distilled water (approx. 20 g) was added to each sample and the mix was homogenized to obtain the liquid phase, which was directly measured in the refractometer. The soluble solid content of the dried fruit was obtained by using the following equations:


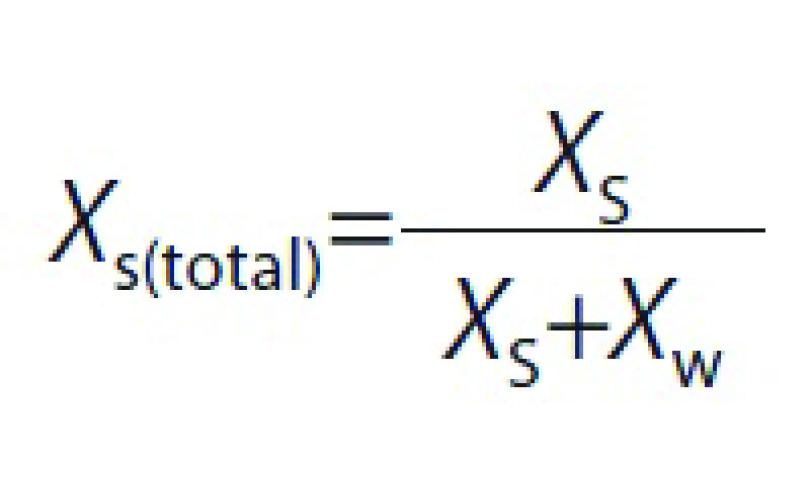



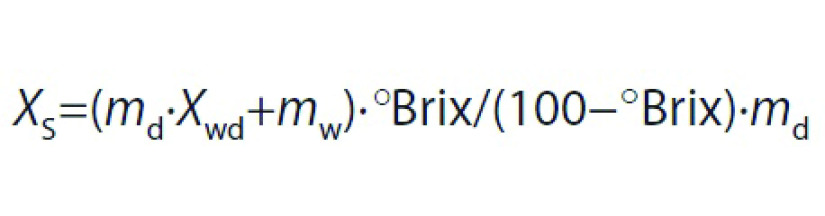


where *X*_s(total)_ is the mass fraction of total soluble solids (in g soluble solids per g fruit liquid phase), *X*_s_ is the mass fraction of soluble solids (in g soluble solids per g fruit), *X*_w_ is a mass fraction of water, *m*_d_ and *m*_w_ are the mass of the dried fruit and the added water used in the analysis, respectively, and *X*_wd_ is the mass fraction of water in the dried fruit.

### Measurement of water activity

A hygrometer (0.003 accuracy; LabMaster, Novasina AG, Lachen, Switzerland) was applied to determine water activity of samples after calibration by K_2_SO_4_ standard solution (*a*_w_=0.972).

### Determination of shrinkage, apparent and bulk densities

Shrinkage (*S*_b_) of samples was estimated by measuring the volume of apricot samples before and after drying. For this purpose, five apricot cubes were selected randomly, and their volume was measured by toluene displacement method. Shrinkage and apparent density (*ρ*_a_) of samples were calculated using the following equations, respectively:


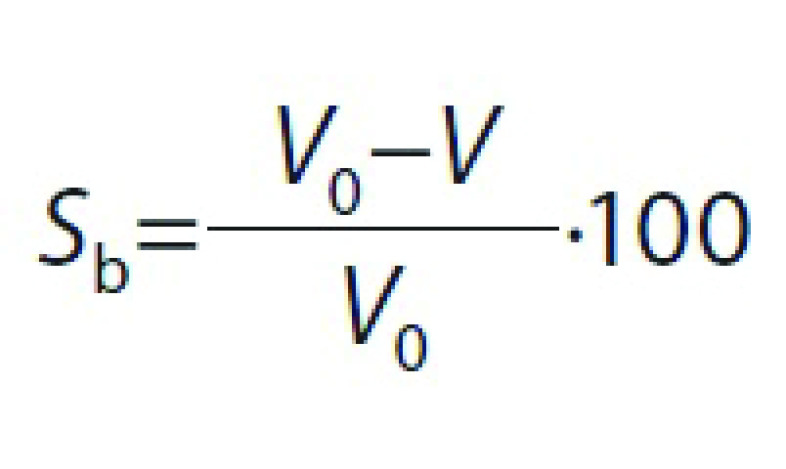



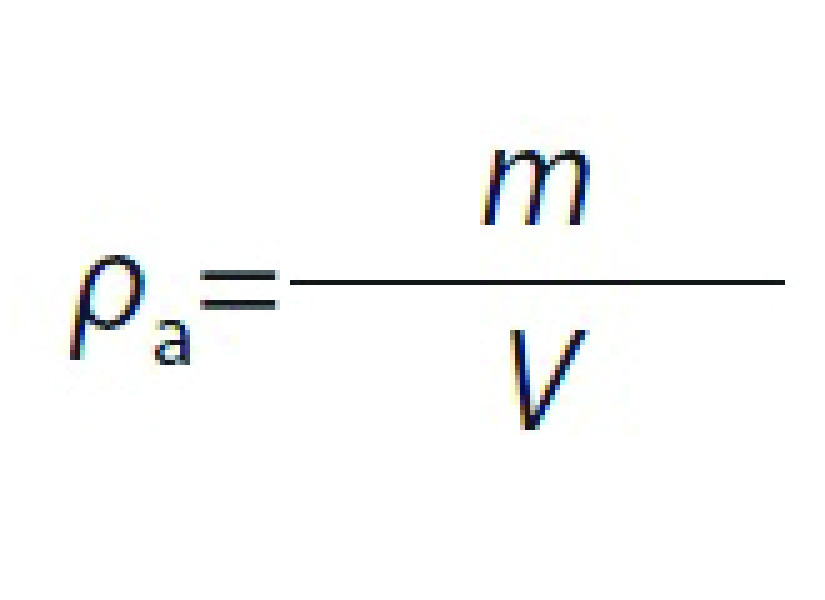


where *S*_b_ is shrinkage (%), *V*_0_ and *V* are the initial and final volumes of apricot (cm^3^), respectively, *ρ*_a_ is the apparent density (g/cm^3^), and *m* is the apricot mass (g).

Bulk density (*ρ*_b_), which is a function of mass fraction of water in samples, was calculated with the following equation:


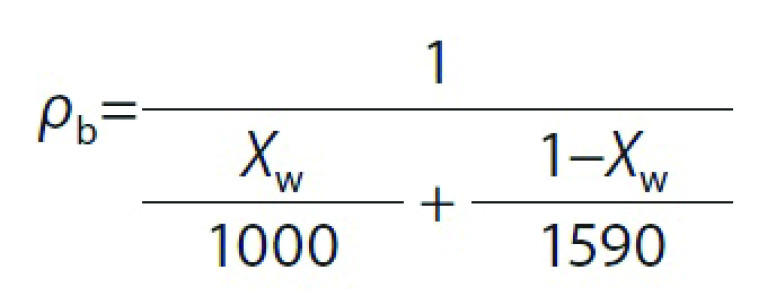


where *ρ*_b_ (g/cm^3^) is the bulk density and *X*_w_ is the mass fraction of water (g/g).

### Determination of rehydration capacity

To measure rehydration capacity, dried samples were weighed and then placed in a glass beaker containing 150 mL distilled water at room temperature for 6 h. Samples were then removed from the distilled water and placed on a paper tissue to eliminate residual water before weighing. Rehydration capacity was calculated as follows (*17*):


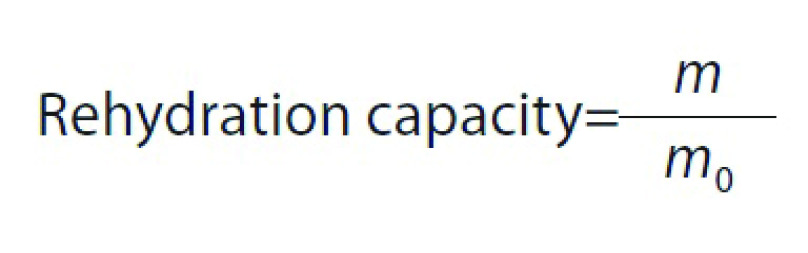


where *m*_0_ and *m* are the initial and final mass of samples (g), respectively.

### Measurement of browning value

Apricot samples were first rehydrated in distilled water, and then rehydration water was clariﬁed by centrifugation at 3200×*g* for 10 min. The supernatant was diluted with an equal volume of 95% ethanol and centrifuged again at 3200×*g* for 10 min. The browning value of the clear extracts was determined in quartz cell using a UV-visible spectrophotometer (Thermo Electron Corporation, Rosemount, MN, USA) at an absorbance of 420 nm (*5*).

### Texture analysis

A texture analyzer (model H5KS; Hounsﬁeld Test Equipment, Redhill, UK) was used to measure the maximum tolerable force, which is related to the ﬁrmness of the dried apricots. The test parameters were set to a pretest speed of 0.1 cm/s, test speed of 0.2 cm/s, distance of 0.3 cm using cylindrical puncture flathead probe with a diameter of 0.2 cm (*18*).

### Microstructure analysis

A scanning electron microscope (XL-30; Philips, Amsterdam, The Netherlands) was used to analyse the microstructural changes after OD and UOD pretreatments. SEM images of freeze-dried samples were obtained after coating of sample strips (thickness 0.1 cm) with a very thin layer of gold under high vacuum (*19*).

### Microbiological analysis

For all microbiological counts, 10 g of sample was aseptically weighed and transferred into 90 mL Ringer’s solution and homogenized. A dilution series of each sample was prepared from 10^-1^ to  10^-6^. The total number of mesophilic aerobic microorganisms was estimated with plate count agar (PCA; Merck KGaA, Darmstadt, Germany). Yeasts and moulds were cultivated and counted with yeast extract glucose chloramphenicol agar (YGC; Merck KGaA). The PCA plates were incubated at 35 °C for two days, whereas YGC plates were incubated at 25 °C for five days. The results of all counts were recorded as the mean value of three -measurements (*2*).

### Experimental design and statistical analysis

In this study, a set of 3×2×3 factorial experiments in a completely randomized way with three replicates were applied. Two ultrasound frequency levels (25 and 35 kHz), two immersion durations (30 and 45 min), and three edible coatings (pectin with citric acid, pectin with ascorbic acid, and only pectin) were used. Physical properties including shrinkage, apparent and bulk densities, texture, microstructure, water activity, microbial load, and browning value were studied. The data obtained from experiments were analysed using Design-Expert software, v. 6.0.1 (*20*). To evaluate the difference between mean values of responses, Duncan’s multiple range test was performed, and significant differences were defined at p<0.05. The Pearson correlation test was also used to determine any correlations among the responses.

## RESULTS AND DISCUSSION

### Hot air drying curves

**Fig. 1:**
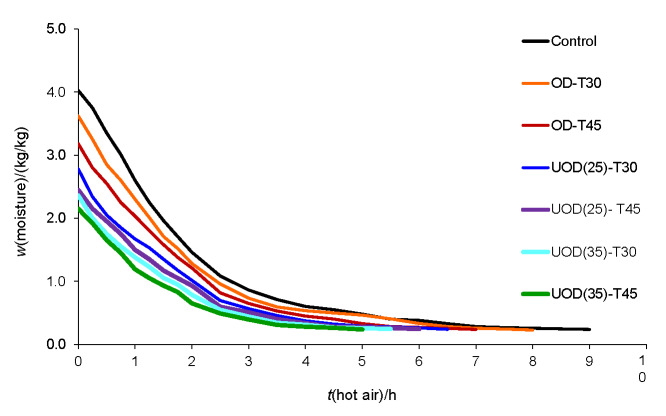
Drying curves of apricot samples treated by osmotic dehydration (OD) for 30 (OD-T30) and 45 min (OD-T45), and ultrasond–assisted osmotic dehydration (UOD) at frequencies of 25 [UOD(25)] and 35 kHz [UOD(35)] for 30 and 45 min

The variation in moisture mass fraction of apricot cubes during hot air drying is shown in Fig. 1. The moisture content of hot air-dried OD-pretreated fresh apricots decreased from 4.0 to an average of 3.4 kg/kg. UOD treatment at 25 kHz and both temperatures reduced the moisture mass fraction to 2.8 kg/kg, while the same treatment at 35 kHz for 45 min caused the highest moisture decrease on dry matter basis (2.3 kg/kg). Control sample required a drying time of 9 h to reach a constant moisture of 0.2 kg/kg, while it took 8 and 7 h for the samples treated by OD for 35 and 45 min, respectively. Drying time was 6 h for samples treated by UOD at 25 kHz and 5 h at 35 kHz at both temperatures. The higher rate of moisture loss of UOD-treated samples was due to the effect of power ultrasound in the formation of fractures and microchannels in the apricot tissue, which enhances drying rate and decreases drying time (*7*).

### Browning value

**Fig. 2:**
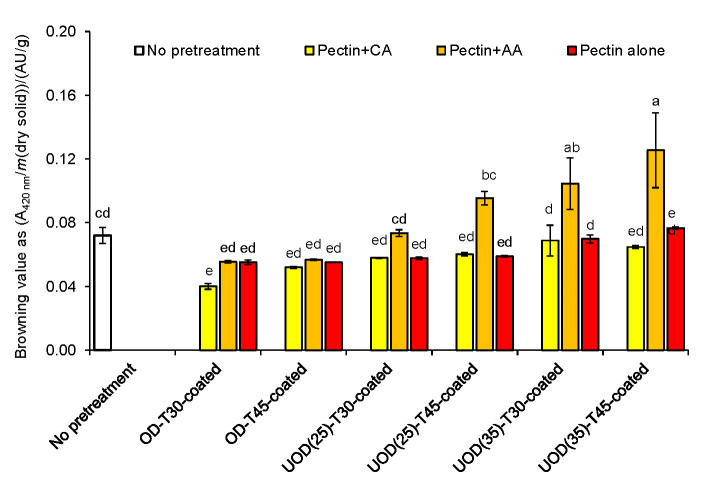
Effect of different osmotic treatments (OD for 30 and 45 min, UOD at 25 and 35 kHz for 30 and 45 min), and the application of pectin, pectin with citric acid (CA), and pectin with acetic acid (AA) coatings on browning values of hot air-dried apricots. Data are mean value of triplicate measurements. Error bars indicate S.D. values. Different letters correspond to significant (p<0.05) differences between mean values. OD=osmotic dehydration, UOD=ultrasound-assisted osmotic dehydration

Fig. 2 represents the effect of OD, UOD and the use of different pectin-based coatings on the browning values of hot air-dried apricots. Application of pectin and pectin with citric acid coatings after the OD treatment reduced browning values in the hot air-dried samples. This can be explained by the effect of OD treatment on the inactivation of polyphenol oxidase enzyme (*21*) and the effect of active coatings in the inhibition of oxidation during hot air drying. We specifically show that pectin with citric acid coating has an effective role on browning inhibition in the OD-treated samples. Moreover, no significant differences (p>0.05) were observed between the browning value of the OD-treated samples coated with either pectin or pectin with acetic acid. Browning values of the UOD-treated samples were higher than of the OD-treated samples. This may be attributed to the effect of power ultrasound in the breakdown of cell walls leading to possible exposure of amino acids and sugars that can participate in the Maillard reaction during drying. For this reason, increasing ultrasound frequency from 25 to 35 kHz increased the browning value the UOD-treated samples. Moreover, UOD-treated samples coated with pectin and ascorbic acid showed higher browning values than samples coated with pectin and citric acid and pectin alone. Oxidation of l-ascorbic acid to l-dehydroascorbic acid and its participation in the Maillard reaction during drying can play a role here (*22*).

**Fig. S1:**
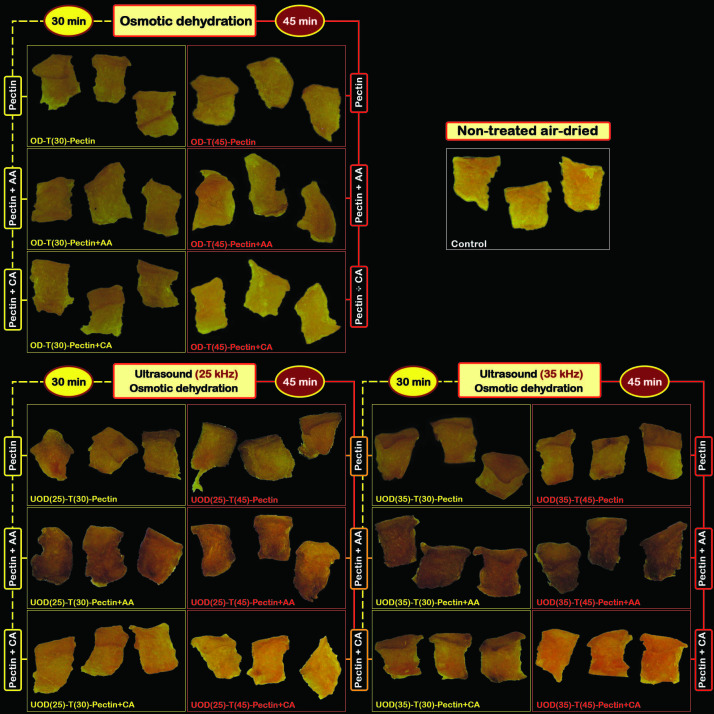
Images of non-treated apricots (control) and samples treated by osmotic dehydration (OD) for 30 and 45 min (T30 and T45), ultrasound-assisted osmotic dehydration (UOD) at frequencies of 25 and 35 kHz for 30 and 45 min (T30 and T45), followed by the application of pectin, pectin with citric acid (CA), and pectin with ascorbic acid (AA) coatings

Fig. S1 shows the browning behaviour of OD- and UOD-treated samples coated with different coatings compared to control. OD-treated samples for 30 or 45 min showed similar browning effect. Likewise, OD samples coated with either pectin or pectin with ascorbic acid had comparable browning behaviour. However, samples treated for 45 min and coated with pectin and citric acid preserved their colour better. Also, UOD samples treated at 35 kHz for 30 or 45 min and coated with pectin and ascorbic acid showed the highest browning effect. The lowest browning effect was observed in the UOD-treated samples with pectin and citric acid coating treated for 30 min. These observations confirm the results presented in Fig. 2.

### Firmness

**Fig. 3:**
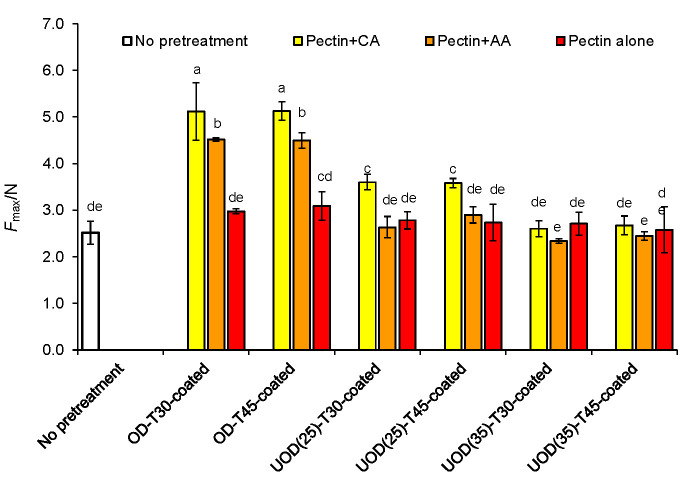
Effect of different osmotic treatments (OD for 30 and 45 min, UOD at 25 and 35 kHz for 30 and 45 min), and the application of pectin, pectin with citric acid (CA), and pectin with ascorbic acid (AA) coatings on maximum force (*F*_max_) of hot air-dried apricots. Data are mean of triplicate measurements. Error bars indicate S.D. values. Different letters represent significant (p<0.05) differences between mean values. OD=osmotic dehydration, UOD=ultrasound-assisted osmotic dehydration

**Fig. 4:**
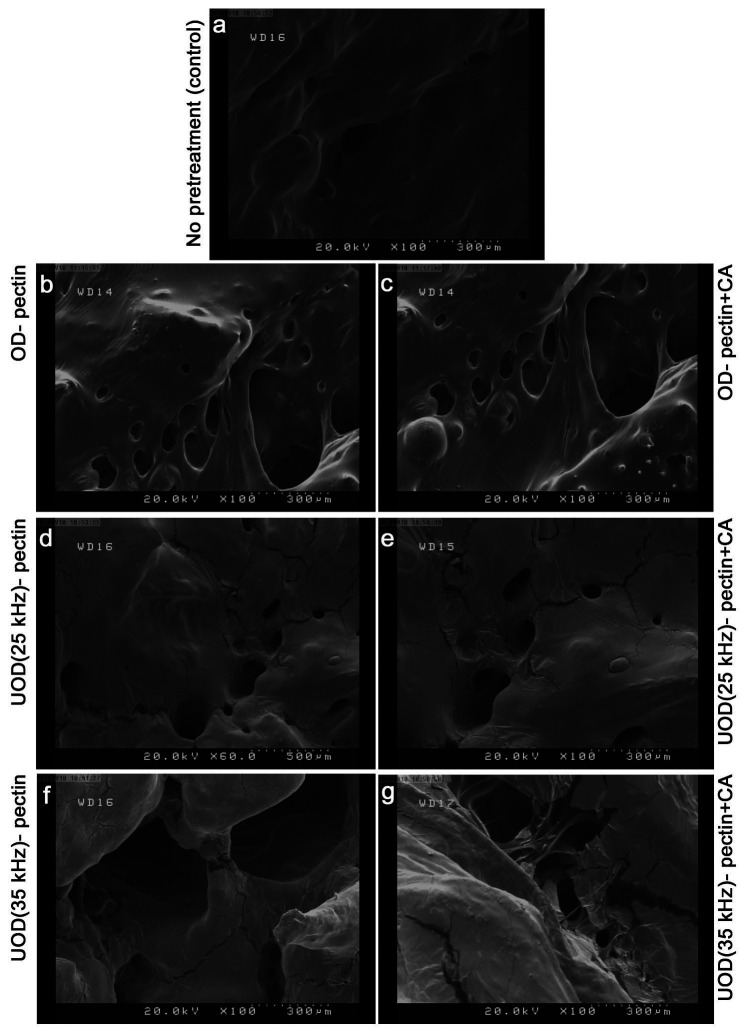
Scanning electron microscopy (SEM) images of: a) control, OD-treated and coated with: b) pectin, c) pectin and citric acid, UOD-treated at 25 kHz and coated with: d) pectin, e) pectin and citric acid, UOD-treated at 35 kHz and coated with: f) pectin, g) pectin and citric acid. OD=osmotic dehydration, UOD=ultrasound-assisted osmotic dehydration

Fig. 3 shows firmness results, expressed as *F*_max_ values, of dried apricot samples treated with OD, UOD and coating (pectin, pectin with citric acid, and pectin with ascorbic acid). OD-treated and coated samples had higher firmness values than control. The results showed that the composition of active coatings significantly (p<0.05) affected *F*_max_ values. There was no significant (p<0.05) difference between the *F*_max_ of control and the OD-coated sample coated with pectin only. However, pectin with citric acid or pectin with acetic acid coatings gave higher *F*_max_ values than pectin only and control samples. This is possibly due to the acidic conditions in pectin coatings containing citric or acetic acid, which influenced the firmness values of these samples. Ben-Shalom *et al*. (*23*) studied the effect of acidiﬁcation following blanching on the ﬁrmness of the carrot tissue. Blanching the carrot tissue at pH=6.2 caused a signiﬁcant reduction (about 70%) in the ﬁrmness of the carrot tissue. For comparison, acidifying and blanching the tissue at pH=4.4 increased (about 50%) the ﬁrmness. Our results also show that OD-treated samples coated with pectin and citric acid had significantly (p<0.05) higher firmness values than that of pectin with acetic acid coating. OD treatment combined with power ultrasound (UOD) led to the firmness decline in these samples. There are two explanations here: (*i*) fractures and microchannels formed by power ultrasound cavitation effect in the apricot tissue caused a decrease of maximum force. These structural changes are seen well in SEM images (Fig. 4). Increase of ultrasound frequency from 25 to 35 kHz led to a significant (p<0.05) decrease in *F*_max_ values of UOD-treated samples. As seen in Fig. 4, larger cavities and fractures were formed in UOD samples treated at 35 kHz. Shamaei *et al.* (*18*) also reported a decrease of *F*_max_ values in air-dried samples by increasing the ultrasound frequency from 35 to 130 kHz in the UOD pretreatment of cranberries; and (*ii*) UOD pretreatment of apricot in sorbitol solutions at 55 °C resulted in more destruction of pectin inside apricot tissue, which causes softer texture for these samples. Xu *et al.* (*24*) reported that the simultaneous application of heat (60 °C) and power ultrasound on grapefruit skin facilitated the extraction of pectin and caused more destruction and depolymerization of pectin molecules. Liu and Zhang (*25*) also reported that application of power ultrasound on citrus pectin decreased the molecular mass of the pectin immediately after the pretreatment with ultrasound waves.

### Microstructural analysis

SEM images of dried apricots are shown in Fig. 4. The image of dried apricot without pretreatment (control) showed destruction of the cell wall and tissue structure collapse. This observation is similar to the reported microstructure of rabbiteye blueberries dried without any pretreatment (*26*). SEM images of OD samples showed swollen inner tissue compared to that of control. Sponge-like tissue formed by cavities, fractures and microchannels were observed in the SEM images of UOD-pretreated samples. Fernandes *et al.* (*27*) found that using ultrasound before drying of pineapple caused more destruction to cellular structure and created microchannels in the internal tissue. Garcia-Noguera *et al.* (*19*) reported that the observed changes in the texture of strawberry after application of UOD were due to the cavitation and the effects of induced osmotic pressure generated by ultrasound waves. Stojanovic and Silva (*26*) reported an extensive collapse in the external surface and cavities, which were distributed uniformly in the internal tissue of the berries pretreated by UOD. As observable in the SEM images in Fig. 4, increasing ultrasound frequency from 25 to 35 kHz created large cavities in the internal tissue of apricot, which were not uniformly distributed. More destruction of inner tissue concurrent with the formation of more microchannels and large cavities have been reported by Shamaei *et al.* (*18*) when the frequency of ultrasound increased from 35 to 130 kHz during UOD retreatment of cranberry. The obtained SEM images showed that pectin coatings with different composition did not affect the microstructure of both OD- and UOD-pretreated apricot samples. Garcia *et al.* (*10*) used TEM imaging to study the microstructure of coated and noncoated dried papaya and reported that the coating itself did not protect the tissue structure from changes during drying.

### Measurement of rehydration capacity

**Fig. 5:**
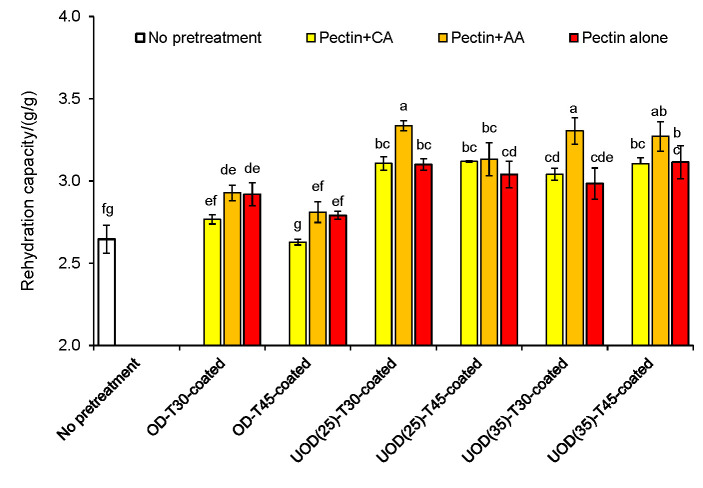
Effect of different osmotic treatments (OD for 30 and 45 min, UOD at 25 and 35 kHz for 30 and 45 min), and the application of pectin, pectin with citric acid (CA), and pectin with ascorbic acid (AA) coatings on rehydration capacity of hot air-dried apricots. Data are mean value of triplicate measurements. Error bars indicate S.D. values. Different letters correspond to significant (p<0.05) differences between mean values. OD=osmotic dehydration, UOD=ultrasound-assisted osmotic dehydration

Fig. 5 shows rehydration capacity of OD- and UOD-pretreated samples coated by pectin, pectin with citric and pectin with ascorbic acid coatings. OD-pretreated and coated samples had slightly higher rehydration capacity than control, although this difference is not significant (p>0.05). Higher rehydration capacity can be attributed to open internal tissue structure of the OD samples, also observable in SEM images (Fig. 4). Erba *et al.* (*28*) showed that the use of sugar alcohols such as sorbitol as the osmotic solution in the pretreatment of fruits could lead to products with good rehydration properties, with possible application in bakery products and ice creams. Application of power ultrasound produced dried apricots with significantly (p<0.05) higher rehydration capacity than of OD-treated and control samples. This can be explained by the formation of fractures, cavities and microchannel in the apricot tissue during UOD pretreatment (Fig. 4). Increasing ultrasound frequency from 25 to 35 kHz had no significant (p>0.05) effect on the rehydration capacity of UOD-pretreated samples. We also show that the coating of the pretreated apricots by different pectin coatings affected the rehydration capacity of the samples. The rehydration capacity of both treated samples coated with pectin and citric acid was significantly (p<0.05) lower than that of coated with pectin and ascorbic acid. These findings are in agreement with those reported by Doymaz (*29*).

Table 1 compares the apparent and bulk densities, and shrinkage values of control sample with those of OD- and UOD-pretreated apricots with different coatings. Control had the lowest apparent and bulk densities, and the highest shrinkage compared to OD- and UOD-pretreated samples. Application of OD before drying with hot air resulted in a significant (p<0.05) increase in bulk and apparent densities. These results are consistent with earlier studies that reported the effect of OD on the increas of the bulk density of hot air-dried apples (*30*, *31*). Udomkun *et al.* (*32*) reported that osmotic treatment of papaya slices before freeze-drying increased apparent density and solid density of the samples. Shrinkage decreased in OD-pretreated and coated samples compared to control. This is attributed to the filling of spaces between the cells by soluble solids of the osmotic solution and reducing any structure collapse in apricot tissue (*33*). SEM images in Fig. 4 display extensively swollen internal tissue in osmotically pretreated samples that can prevent shrinkage during hot air drying. Reppa *et al.* (*34*) found that osmotic pretreatment of apple slices decreased the shrinkage of dried samples, and there is a direct relationship between the reduction of shrinkage and mass transfer of soluble solids from the osmotic solution to fruit tissue. Table 1 shows that the UOD pretreatment significantly (p<0.05) reduced shrinkage and increased apparent and bulk densities of dried apricots. The created cavities and microchannels in the tissue of UOD-pretreated samples (Fig. 4) facilitate penetration of soluble solids from osmotic solution into the tissue during UOD pretreatment. Moreover, these structural changes enhance drying speed, and therefore, decrease the shrinkage of UOD-pretreated samples during hot air drying. This effect was even more enhanced when ultrasound frequency increased from 25 to 35 kHz. Stojanovic and Silva (*26*) reported an increase in the bulk density of UOD-pretreated rabbiteye blueberries. UOD-pretreated samples treated at 35 kHz for 45 min had the lowest shrinkage and the highest apparent density values. Samples with higher bulk density had higher rehydration capacity and lower shrinkage values.

**Table 1:** Variation of bulk (*ρ*_b_) and apparent (*ρ*_a_) densities, and shrinkage (*S*_b_) in non-treated (control) and air-dried apricots by different pretreatments: osmotic dehydration (OD) for 30 and 45 min, ultrasound-assisted osmotic dehydration (UOD) at 25 and 35 kHz for 30 and 45 min, and application of coatings consisting of pectin only (P), pectin with citric acid (CA), and pectin with ascorbic acid (AA)

Pretreatment	*ρ*_b_/(g/cm^3^)	*ρ*_a_/(g/cm^3^)	*S*_b_/%
Dried, non-treated (control)	(1.358±0.000)^g^	(1.260±0.000)^g^	(84.5±0.9)^f^
OD-T30-coated (P+CA)	(1.368±0.002)^f^	(1.266±0.002)^efg^	(83.2±0.1)^ef^
OD-T30-coated (P+AA)	(1.366±0.000)^f^	(1.262±0.004)^fg^	(83.1±0.2)^ef^
OD-T30-coated (P)	(1.366±0.000)^f^	(1.259±0.003)^g^	(83.0±0.5)^ef^
OD-T45-coated (P+CA)	(1.367±0.000)^f^	(1.268±0.002)^defg^	(80.4±0.7)^abcd^
OD-T45-coated (P+AA)	(1.367±0.000)^f^	(1.268±0.000)^defg^	(81.1±0.3)^bcde^
OD-T45-coated (P)	(1.367±0.000)f	(1.268±0.001)^defg^	(81.3±1.0)^cde^
UOD(25)-T30-coated (P+CA)	(1.405±0.000)^d^	(1.279±0.002)^bcdef^	(82.4±1.6)^de^
UOD(25)-T30-coated (P+AA)	(1.399±0.020)^e^	(1.273±0.002)^cdefg^	(81.5±0.7)^cde^
UOD(25)-T30-coated (P)	(1.403±0.000)^cd^	(1.279±0.003)^bcdef^	(81.8±0.5)^cde^
UOD(25)-T45-coated (P+CA)	(1.404±0.002)^bcd^	(1.283±0.001)^bcde^	(79.9±1.9)^abc^
UOD(25)-T45-coated (P+AA)	(1.402±0.002)^d^	(1.283±0.000)^bcde^	(81.2±0.10)^cde^
UOD(25)-T45-coated (P)	(1.405±0.000)^abcd^	(1.286±0.007)^abc^	(81.0±0.3)^bcde^
UOD(35)-T30-coated (P+CA)	(1.404±0.001)^bcd^	(1.283±0.004)^bcde^	(81.6±0.5)^cde^
UOD(35)-T30-coated (P+AA)	(1.405±0.002)^abcd^	(1.284±0.003)^abcd^	(80.6±0.8)^abcd^
UOD(35)-T30-coated (P)	(1.405±0.002)^abcd^	(1.284±0.009)^bcd^	(80.6±0.8)^abcd^
UOD(35)-T45-coated (P+CA)	(1.407±0.000)^a^	(1.301±0.013)^a^	(79.0±1.0)^ab^
UOD(35)-T45-coated (P+AA)	(1.407±0.000)^ab^	(1.296±0.006)^ab^	(79.0±1.3)^ab^
UOD(35)-T45-coated (P)	(1.406±0.000)^abc^	(1.291±0.006)^ab^	(78.7±1.2)^a^

Data are mean value of triplicate measurements±standard deviation. Different letters in each column represent significant (p<0.05) differences between mean values

Table 2 shows the average values of water and soluble solid content of the fresh and pretreated dried apricot samples. The initial water mass fraction decreased from 82 to 21-27 g/100 g fruit in pretreated dried apricots. Therefore, the soluble solid content increased from 18 to around 79-73 g/100 g fruit liquid phase in these samples. The water activity was 0.929 in fresh samples, which decreased to 0.628 in non-treated dried apricots. Sugar content increase due to osmotic dehydration (with or without ultrasound) affected the water activity in the pretreated dried apricots. Water activity was within the range of 0.547-0.560 in OD-pretreated and coated samples. The formed cavities and microchannels in the UOD-pretreated apricot tissue (Fig. 4) increased sugar content and accelerated water loss during UOD pretreatment. Table 2 shows that with the increase of the mass fraction of soluble solids from 0.641-0.644 g/g in the OD-pretreated samples to 0.672-0.724 g/g in the UOD-pretreated ones sugar content in the UOD-pretreated samples increased. Increase in sugar gain, significantly affected water activity in these samples as it was lower in the UOD-pretreated samples than in the OD-pretreated ones. Kowalski *et al.* (*35*) reported that ultrasound-assisted osmotic dehydration in combination with intermittent-convective drying of cherry caused lower water activity. Also, an increase of ultrasound frequency from 25 to 35 kHz significantly (p<0.05) increased the mass fraction of soluble solids in these samples and led to significant (p<0.05) reduction in water activity of the UOD-treated apricots. The water activity of the UOD-pretreated and coated apricots was 0.546-0.522. The samples treated at 35 kHz had the lowest water activity. Shamaei *et al.* (*18*) indicated that increasing the ultrasound frequency from 35 to 130 kHz in ultrasound-assisted osmotic dehydration of cranberry decreased the water activity of the dried cranberries. Statistical analysis indicated that coatings and their compositions did not have any significant (p>0.05) effects on the water activity of dried apricots. Immersion duration in both OD- and UOD-pretreated samples did not change the water activity of dried samples significantly.

**Table 2:** Variation of the mass fraction of soluble solids (*X*_s_), mass fraction of water (*X*_w_) and water activity (*a*_w_) in non-treated (control) and air-dried apricots by different pretreatments: osmotic dehydration (OD) for 30 and 45 min, ultrasound-assisted osmotic dehydration (UOD) at 25 and 35 kHz for 30 and 45 min, and application of coatings consisting of pectin only (P), pectin with citric acid (CA), and pectin with ascorbic acid (AA)

Pretreatment	*X*_s_(*m*(soluble solid)/*m*(fruit) /(g/g)	*X*_w_(m(water)/m(fruit))/(g/g)	*a*_w_
Fresh apricot	(0.186±0.002)^k^	(0.824±0.000)^a^	(0.929±0.003)^e^
Dried (no pretreatment)	(0.622±0.000)^i^	(0.294±0.000)b	(0.628±0.004)^d^
OD-T30-coated (P+CA)	(0.641±0.001)^h^	(0.275±0.002)^c^	(0.548±0.000)^b^
OD-T30-coated (P+AA)	(0.642±0.001)^h^	(0.278±0.000)^c^	(0.561±0.003)^c^
OD-T30-coated (P)	(0.642±0.000)^h^	(0.278±0.000)^c^	(0.549±0.002)^b^
OD-T45-coated (P+CA)	(0.643±0.000)^h^	(0.277±0.000)^c^	(0.547±0.001)^b^
OD-T45-coated (P+AA)	(0.644±0.001)^h^	(0.276±0.000)^c^	(0.548±0.000)^b^
OD-T45-coated (P)	(0.643±0.000)^h^	(0.277±0.000)^c^	(0.549±0.005)^b^
UOD(25)-T30-coated (P+CA)	(0.672±0.007)^g^	(0.227±0.007)^e^	(0.531±0.001)^a^
UOD(25)-T30-coated (P+AA)	(0.681±0.003)^f^	(0.232±0.003)^d^	(0.546±0.001)^b^
UOD(25)-T30-coated (P)	(0.682±0.002)f	(0.226±0.002)^ef^	(0.541±0.002)^b^
UOD(25)-T45-coated (P+CA)	(0.703±0.006)^de^	(0.225±0.006)^ef^	(0.540±0.002)^b^
UOD(25)-T45-coated (P+AA)	(0.698±0.007)^e^	(0.227±0.007)^e^	(0.542±0.003)^b^
UOD(25)-T45-coated (P)	(0.695±0.009)^e^	(0.223±0.010) ^efg^	(0.542±0.005)^b^
UOD(35)-T30-coated (P+CA)	(0.716±0.007)^abc^	(0.225±0.007)^efg^	(0.527±0.002)^a^
UOD(35)-T30-coated (P+AA)	(0.713±0.010)^bc^	(0.223±0.010)^efg^	(0.522±0.004)^a^
UOD(35)-T30-coated (P)	(0.708±0.005)^cd^	(0.223±0.005)^efg^	(0.526±0.008)^a^
UOD(35)-T45-coated (P+CA)	(0.724±0.010)^a^	(0.220±0.000)^h^	(0.531±0.001)^a^
UOD(35)-T45-coated (P+AA)	(0.720±0.002)^ab^	(0.220±0.002)^gh^	(0.529±0.009)^a^
UOD(35)-T45-coated (P)	(0.722±0.000)^a^	(0.222±0.000)^fgh^	(0.531±0.002)^a^

Data are mean value of triplicate measurements±standard deviation. Different letters in each column represent significant (p<0.05) differences between mean values

Table 3 demonstrates the microbial load of nontreated and OD/UOD/coating-treated air-dried apricots. The total mesophilic aerobic count and total yeast and mould count in nontreated air-dried apricots was 115 and 40 CFU/g, respectively. Table 3 shows that the total mesophilic aerobic count and yeast and mould counts reduced significantly (p<0.05) in the OD-pretreated and coated dried apricots. This reduction can be attributed to a reduced water activity of these samples (Table 2). It is known that at the water activity less than 0.6, the activity of bacteria, moulds and yeasts is very low (*8*, *35*). Water activity in control sample was 0.629, which was reduced to 0.547-0.560 in the OD-pretreated and coated samples. Indeed, this reduction provided a substantial decrease in the microbial load of these samples. Also, the evaluation of microbial load in the UOD-pretreated and coated dried apricots indicated that the UOD pretreatment had a positive effect on the microbial load reduction in these samples compared to the OD pretreatment. The total mesophilic aerobic count decreased by 0.29-0.52 log cycles in the UOD-pretreated and coated dried samples compared to the control. Villalobos *et al*. (*36*) reported that UOD-pretreated figs had the lowest microbial load compared to traditionally dried samples. UOD-treated apricot samples at 35 kHz for 45 min had the lowest total mesophilic aerobic count and total yeast and mould count. There was no significant (p<0.05) difference between the microbial load of dried apricots treated with OD or UOD- and coated with three different coatings.

**Table 3:** Microbial load of non-treated (control) and air-dried apricots by different pretreatments: osmotic dehydration (OD) for 30 and 45 min, ultrasound-assisted osmotic dehydration (UOD) at 25 and 35 kHz for 30 and 45 min, and application of coatings consisting of pectin only (P), pectin with citric acid (CA), and pectin with ascorbic acid (AA)

Pretreatment	*N*(total mesophyllic aerobic count) /(CFU/g)	*N*(total yeast and mould)/ (CFU/g)
Dried (No pre-treatment)	(115±7)^a^	(40±7)^a^
OD-T30-coated (P+CA)	(65±7)^cde^	(25±0)^cd^
OD-T30-coated (P+AA)	(75±7)^bc^	(30±7)^bc^
OD-T30-coated (P)	(85±7)^b^	(35±7)^ab^
OD-T45-coated (P+CA)	(60±1)^def^	(20±0)^de^
OD-T45-coated (P+AA)	(70±14)^cd^	(20±14)^def^
OD-T45-coated (P)	(75±7)^bc^	(20±7)^de^
UOD(25)-T30-coated (P+CA)	(50±0)^fgh^	(10±0)^h^
UOD(25)-T30-coated (P+AA)	(55±7)^efg^	(15±0)^fg^
UOD(25)-T30-coated (P)	(60±0)^def^	(15±0)^fg^
UOD(25)-T45-coated (P+CA)	(45±7)^ghi^	(10±7)^hi^
UOD(25)-T45-coated (P+AA)	(50±0)^fgh^	(10±0)^h^
UOD(25)-T45-coated (P)	(55±7)^efg^	(15±7)^fgh^
UOD(35)-T30-coated (P+CA)	(40±0)^hi^	(0±0)^j^
UOD(35)-T30-coated (P+AA)	(45±7)^ghi^	(0±0)^j^
UOD(35)-T30-coated (P)	(50±0)^fgh^	(5±3.5)^i^
UOD(35)-T45-coated (P+CA)	(35±7)^i^	(0±0)^j^
UOD(35)-T45-coated (P+AA)	(40±0)^hi^	(0±0)^j^
UOD(35)-T45-coated (P)	(50±0)^fgh^	(0±0)^j^

Data are mean value of duplicate measurements±standard deviation. Different letters in each column represent significant (p<0.05) differences between mean values

## CONCLUSIONS

The application of ultrasound-assisted osmotic dehydration (UOD) pre-treatment in sorbitol solutions before hot air drying of apricots resulted in the improvement of physical properties such as shrinkage, apparent density, bulk density, rehydration capacity and texture of the dried apricots. When ultrasound frequency was increased from 25 to 35 kHz, firmness decreased, and rehydration capacity increased. While the UOD of apricot samples increased the browning value of the air-dried apricots, the osmotic dehydration (OD) resulted in better colour preservation. After increasing ultrasound frequency from 25 to 35 kHz, discoloration increased in the UOD-pretreated samples. Coating of both OD- and UOD-pretreated samples with pectin with citric acid increased firmness and decreased rehydration capacity. Application of UOD pre-treatment led to water activity and microbial load reduction.

## References

[1] Saracoglu S Tuzen M Soylak M Evaluation of trace element contents of dried apricot samples from Turkey. J Hazard Mater. 2009;167(1-3):647–52. 10.1016/j.jhazmat.2009.01.011 19195780

[2] Karabulut I Topcu A Duran A Turan S Ozturk B Effect of hot air drying and sun drying on color values and β-carotene content of apricot (*Prunus armenica* L.). Lebensm Wiss Technol. 2007;40(5):753–8. 10.1016/j.lwt.2006.05.001

[3] Albanese D Cinquanta L Cuccurullo G Di Matteo M Effects of microwave and hot-air drying methods on colour, β-carotene and radical scavenging activity of apricots. Int J Food Sci Technol. 2013;48(6):1327–33. 10.1111/ijfs.12095

[4] Altındağ M Türkyılmaz M Özkan M Changes in polyphenol profile of dried apricots containing SO_2_ at various concentrations during storage. J Sci Food Agric. 2018;98(7):2530–9. 10.1002/jsfa.8740 29023817

[5] Hamzaoğlu F Türkyilmaz M özkan M. Amino acid profile and content of dried apricots containing SO_2_ at different concentrations during storage. Qual Assur Saf Crops Foods. 2018;10(4):361–9. 10.3920/QAS2018.1284

[6] Lemus-Mondaca R Miranda M Andres Grau A Briones V Villalobos R Vega-Gálvez A Effect of osmotic pretreatment on hot air drying kinetics and quality of Chilean papaya (*Carica pubescens*). Dry Technol. 2009;27(10):1105–15. 10.1080/07373930903221291

[7] Çağlayan D Barutçu Mazı I Effects of ultrasound-assisted osmotic dehydration as a pretreatment and finish drying methods on the quality of pumpkin slices. J Food Process Preserv. 2018;42(9):e13679 10.1111/jfpp.13679

[8] Bromberger Soquetta M Schmaltz S Wesz Righes F Salvalaggio R de Marsillac Terra L Effects of pretreatment ultrasound bath and ultrasonic probe, in osmotic dehydration, in the kinetics of oven drying and the physicochemical properties of beet snacks. J Food Process Preserv. 2018;42(1):e13393 10.1111/jfpp.13393

[9] Silva KS Garcia CC Amado LR Mauro MA Effects of edible coatings on convective drying and characteristics of the dried pineapple. Food Bioprocess Technol. 2015;8:1465–75. 10.1007/s11947-015-1495-y

[10] Garcia CC Caetano LC de Souza Silva K Mauro MA Influence of edible coating on the drying and quality of papaya (*Carica papaya*). Food Bioprocess Technol. 2014;7:2828–39. 10.1007/s11947-014-1350-6

[11] Ghasemzadeh R Karbassi A Ghoddousi HB Application of edible coating for improvement of quality and shelf-life of raisins. World Appl Sci J. 2008;3(1):82–7.

[12] Cheng XF Zhang M Adhikari B The inactivation kinetics of polyphenol oxidase in mushroom (*Agaricus bisporus*) during thermal and thermosonic treatments. Ultrason Sonochem. 2013;20(2):674–9. 10.1016/j.ultsonch.2012.09.012 23102768

[13] Tortoe C. A review of osmodehydration for food industry. Afr J Food Sci. 2010;4(6):303–24. 10.5897/AJFS.9000007

[14] Fernandes FAN Gallão MI Rodrigues S Effect of osmotic dehydration and ultrasound pre-treatment on cell structure: Melon dehydration. Lebensm Wiss Technol. 2008;41(4):604–10. 10.1016/j.lwt.2007.05.007

[15] Deng Y Zhao Y Effect of pulsed vacuum and ultrasound osmopretreatments on glass transition temperature, texture, microstructure and calcium penetration of dried apples (Fuji). Lebensm Wiss Technol. 2008;41(9):1575–85. 10.1016/j.lwt.2007.10.018

[16] Official Method AOAC. 927.05-1927. Moisture in dried milk. Rockville, MD, USA: AOAC International; 2011.

[17] Taiwo KA Angersbach A Knorr D Rehydration studies on pretreated and osmotically dehydrated apple slices. J Food Sci. 2002;67(2):842–7. 10.1111/j.1365-2621.2002.tb10687.x

[18] Shamaei S Emam-Djomeh Z Moini S Ultrasound-assisted osmotic dehydration of cranberries: Effect of finish drying methods and ultrasonic frequency on textural properties. J Texture Stud. 2012;43(2):133–41. 10.1111/j.1745-4603.2011.00323.x

[19] Garcia-Noguera J Oliveira FIP Gallão MI Weller CL Rodrigues S Fernandes FAN Ultrasound-assisted osmotic dehydration of strawberries: Effect of petreatment time and ultrasonic frequency. Dry Technol. 2010;28(2):294–303. 10.1080/07373930903530402

[20] Design-Expert, v. 6.0.1, Stat-Ease Inc., Minneapolis, MN, USA; 2017. Available from: www.statease.com/software/design-expert.

[21] Riva M Campolongo S Leva AA Maestrelli A Torreggiani D Structure-property relationships in osmo-air-dehydrated apricot cubes. Food Res Int. 2005;38(5):533–42. 10.1016/j.foodres.2004.10.018

[22] Pischetsrieder M, Larisch B, Severin T. The Maillard reaction of ascorbic acid with amino acids and proteins - Identification of Products. In: O’Brien J, Nursten HE, Crabbe MJC, Ames JM, editors. The Maillard reaction in foods and medicine. Series in Food Science, Technology and Nutrition. Woodhead Publishing; 2005. pp. 107–12. https://doi.org/10.1533/9781845698447.2.107

[23] Ben-Shalom N Plat D Levi A Pinto R Changes in molecular weight of water-soluble and EDTA-soluble pectin fractions from carrot after heat treatments. Food Chem. 1992;45(4):243–5. 10.1016/0308-8146(92)90154-T

[24] Xu Y Zhang L Bailina Y Ge Z Ding T Ye X Effects of ultrasound and/or heating on the extraction of pectin from grapefruit peel. J Food Eng. 2014;126:72–81. 10.1016/j.jfoodeng.2013.11.004

[25] Liu D Zhang L The influence of ultrasound on the structure, rheological properties, and degradation path of citrus pectin. J Acoust Soc Am. 2013;133(5):3595. 10.1121/1.480664823654363

[26] Stojanovic J Silva JL Influence of osmoconcentration, continuous high-frequency ultrasound and dehydration on properties and microstructure of rabbiteye blueberries. Dry Technol. 2006;24(2):165–71. 10.1080/07373930600558995

[27] Fernandes FAN Gallão MI Rodrigues S Effect of osmosis and ultrasound on pineapple cell tissue structure during dehydration. J Food Eng. 2009;90(2):186–90. 10.1016/j.jfoodeng.2008.06.021

[28] Erba ML Forni E Colonello A Giangiacomo R Influence of sugar composition and air dehydration levels on the chemical-physical characteristics of osmodehydrofrozen fruit. Food Chem. 1994;50(1):69–73. 10.1016/0308-8146(94)90095-7

[29] Doymaz İ Effect of citric acid and blanching pre-treatments on drying and rehydration of Amasya red apples. Food Bioprod Process. 2010;88(2-3):124–32. 10.1016/j.fbp.2009.09.003

[30] Nieto AB Salvatori DM Castro MA Alzamora SM Structural changes in apple tissue during glucose and sucrose osmotic dehydration: Shrinkage, porosity, density and microscopic features. J Food Eng. 2004;61(2):269–78. 10.1016/S0260-8774(03)00108-0

[31] Krokida MK Maroulis ZB Effect of drying method on shrinkage and porosity. Dry Technol. 1997;15(10):2441–58. 10.1080/07373939708917369

[32] Udomkun P Argyropoulos D Nagle M Mahayothee B Oladeji AE Müller J Changes in microstructure and functional properties of papaya as affected by osmotic pre-treatment combined with freeze-drying. J Food Meas Charact. 2018;12:1028–37. 10.1007/s11694-018-9718-3

[33] Yaǧcı S Göǧüş F Response surface methodology for evaluation of physical and functional properties of extruded snack foods developed from food-by-products. J Food Eng. 2008;86(1):122–32. 10.1016/j.jfoodeng.2007.09.018

[34] Reppa A Mandala J Kostaropoulos AE Saravacos GD Influence of solute temperature and concentration on the combined osmotic and air drying. Dry Technol. 1999;17(7-8):1449–58. 10.1080/07373939908917627

[35] Kowalski SJ Szadzińska J Pawłowski A Ultrasonic-assisted osmotic dehydration of carrot followed by convective drying with continuous and intermittent heating. Dry Technol. 2015;33(13):1570–80. 10.1080/07373937.2015.1012265

[36] Villalobos MC Serradilla MJ Martín A Ruíz-Moyano S Casquete R Hernández A Use of efficient drying methods to improve the safety and quality of dried fig. J Food Process Preserv. 2019;43(1):e13853 10.1111/jfpp.13853

